# Spontaneous local membrane curvature induced by transmembrane proteins

**DOI:** 10.1016/j.bpj.2022.01.029

**Published:** 2022-02-03

**Authors:** Christoph Kluge, Matthias Pöhnl, Rainer A. Böckmann

**Affiliations:** 1 Computational Biology, Department of Biology, Friedrich-Alexander-Universität Erlangen-Nürnberg (FAU), Erlangen, Germany; 2 National Center for High-Performance Computing Erlangen (NHR@FAU), Erlangen, Germany

## Abstract

The (local) curvature of cellular membranes acts as a driving force for the targeting of membrane-associated proteins to specific membrane domains, as well as a sorting mechanism for transmembrane proteins, e.g., by accumulation in regions of matching spontaneous curvature. The latter measure was previously experimentally employed to study the curvature induced by the potassium channel KvAP and by aquaporin AQP0. However, the direction of the reported spontaneous curvature levels as well as the molecular driving forces governing the membrane curvature induced by these integral transmembrane proteins could not be addressed experimentally.

Here, using both coarse-grained and atomistic molecular dynamics (MD) simulations, we report induced spontaneous curvature values for the homologous potassium channel Kv 1.2/2.1 Chimera (KvChim) and AQP0 embedded in unrestrained lipid bicelles that are in very good agreement with experiment. Importantly, the direction of curvature could be directly assessed from our simulations: KvChim induces a strong positive membrane curvature (≈0.036 nm^−1^) whereas AQP0 causes a comparably small negative curvature (≈−0.019 nm^−1^).

Analyses of protein-lipid interactions within the bicelle revealed that the potassium channel shapes the surrounding membrane via structural determinants. Differences in shape of the protein-lipid interface of the voltage-gating domains between the extracellular and cytosolic membrane leaflets induce membrane stress and thereby promote a protein-proximal membrane curvature. In contrast, the water pore AQP0 displayed a high structural stability and an only faint effect on the surrounding membrane environment that is connected to its wedge-like shape.

## Significance

The induction of membrane curvature is required in a vast number of biological processes, ranging from curvature-driven sorting of proteins or lipids to shaping of distinct cellular regions involved, e.g., in signaling or in processes necessitating a remodeling of the cellular shape, e.g., in fusion or scission. Here, we show for two transmembrane channel proteins using molecular dynamics simulations how protein shape affects membrane curvature, report that the interaction radius for membrane remodeling by proteins extends to ≈2 nm from the protein surface, and suggest lipid bicelle systems as a beneficial in silico system for the unbiased determination of protein-induced membrane curvature.

## Introduction

Membrane-embedded proteins are not equally distributed between and within the different (organelle) membranes of cells (1,2). This functionally important inhomogeneity is maintained by targeted protein-partitioning processes between and diffusion within the various membrane types found in cells, both on larger and smaller scales (3). For example, the maturation of eukaryotic plasma membrane proteins involves the active transport from the endoplasmic reticulum and the Golgi apparatus to the plasma membrane via transport vesicles (4). This pathway, a *global* partitioning mechanism of its own, depends on *local* sorting events, such as the actively mediated budding of transport vesicles and the final localization of proteins at specific patterns within the target membrane. In the last years, it was demonstrated that this concerted organization of proteins, in addition to direct protein-protein (5,6) and protein-lipid binding (7,8), can as well be regulated by the utilization of membrane properties as sorting cues, such as membrane thickness (9) or spontaneous curvature (10, 11, 12).

Membrane-thickness-related organization relies on the composition-dependent formation of membrane micro- or nanodomains or lipid rafts with different fluidities and different lipid ordering. The resulting hydrophobic mismatch, i.e., the difference between the length of the hydrophobic transmembrane (TM) segment of proteins and the thickness of the membrane hydrophobic core, guides proteins into membrane areas of minimal mismatch (13, 14, 15). This mechanism is primarily attributed to integral proteins or proteins anchored by a TM helix.

The spontaneous local or global membrane curvature is linked to the lipid composition as well, similar to the hydrophobic mismatch. However, rather than depending on the lipid tail length, curvature stress emerges from the different head-to-tail-size ratios of the various lipid species found in biological systems and their distribution between the bilayer leaflets (16, 17, 18, 19). Additional protein-membrane-shaping machineries and cytoskeleton actin filaments (20, 21, 22, 23, 24) further differentiate the morphology, which can range from specifically folded structures, such as organelle membranes (25, 26, 27, 28, 29, 30, 31, 32), to small spherical transport vesicles and membrane tubuli (33, 34, 35, 36).

Recent research uncovered that several membrane-associated proteins and peptides are able to bind to the membrane by fitting inherent curvature (37, 38, 39, 40) or by sensing of lipid packing defects in positively curved membranes (41, 42, 43). The former, adsorption to matching curvature, is prominently displayed by the crescent-shaped, “Bin/Amphiphysin/Rvs” (BAR) protein family (44). The observed structural “curvature scaffold” allows BAR-domain proteins to detect patches of fitting curvature and sorting copies of the respective BAR-domain proteins to pre-existing membrane buds or comparably shaped membranes. Multiple variants of the basic BAR-domain structure were described during the last years, with scaffolds ranging from highly positive curvature (Arfaptin (45)) to negative curvature (IRSp53 (46)). The latter, detection of packing defects, depends on amphipathic α helices (43,47) or specialized amphipathic lipid packing sensor (ALPS) motifs (48, 49, 50), which contain large hydrophobic amino acids, which dynamically bind to packing defects of fitting size as they emerge from increasing positive curvature (43,51). Special cases observed in bacterial cells include the binding to negatively curved membranes (52,53) as well as to very shallowly curved membranes (54). Interestingly, based on an entropic mechanism, intrinsically disordered regions in proteins were shown to amplify the membrane curvature sensitivity to convex membrane surfaces (55).

However, also TM proteins may induce curvature stress in the surrounding membrane and thereby sort themselves into correspondingly shaped membrane domains (56, 57, 58, 59), curved by the local (asymmetric) lipid composition or external shaping. Coined “spontaneous curvature mismatch,” this principle is supported by cryoelectron microscopy experiments (60) that showed ring- or tube-like aggregates for the bacterial ABC transporter BmrA and pointed to an asymmetric distribution of lipids around the protein's TM domain, leading to membrane curvature and eventually ring formation. Strahl et al. (61) further showed that, in vivo, the chemoreceptor TlpA of bacterium *B*. *subtilis* sorts itself to highly curved regions during cell division. Most interestingly, the study suggests that this behavior is solely based on the conical shape of the functional complex formed by a trimer of dimers, since inhibited trimerization or increased trimer flexibility led to a diffuse protein distribution. Similarly, a correlation between receptor shape and membrane curvature was reported for the ligand-regulated sorting of G-protein-coupled receptors (58).

In the in vitro comparison study of Aimon et al. (62), the curvature-dependent distribution for two integral TM model proteins was investigated using lipid nanotubes of controllable diameter. The bacterial potassium channel KvAP (63) was shown to be enriched in nanotubes with radii between 15 nm and 35 nm, which translates into a preferred spontaneous curvature of ≈0.04 nm^−1^. In turn, the distribution of the aquaporin water channel (AQP0) (64) did not change significantly with decreasing radii while settling at a membrane curvature of less than 0.02 nm^−1^. Notably, the sign of the spontaneous curvature values retrieved from experiment, i.e., the insertion direction of the investigated proteins, was not accessible from the performed protocol. A second experimental study performed by Quemeneur et al. (65) investigated the diffusion of KvAP and AQP0 as a function of the surrounding membrane tension. From the retrieved data and the applied model, a different spontaneous curvature of KvAP, ≈0.16 nm^−1^, was deduced. Thus, a fourfold discrepancy between the two experimentally retrieved spontaneous KvAP-induced curvatures was established. The trends in curvature induced by AQP0 and KvAP could recently be reproduced in coarse-grained simulations for different membrane strains (66).

Here, combining coarse-grained (CG) and all-atom (AA) molecular dynamics simulations, we established a workflow to study the spontaneous curvature induced by the voltage-gated potassium channel Kv 1.2/2.1 Chimera (KvChim) (67), a functional homologue of KvAP and of AQP0 on the nanometer scale employing lipid bicelles. The chimera potassium channel was chosen because it was crystallized in a lipid-membrane-like environment. The magnitude of induced curvature as well as the direction of curvature are directly accessible from our simulations and strongly support experimental values retrieved from sorting experiments (62). The induced curvature values were further rationalized by analysis of the nanodomain environment of the channels.

## Materials and methods

### Molecular dynamics simulations

For the study of protein-induced membrane curvature, the studied KvChim and AQP0 proteins were embedded in a simple membrane built of 1-palmitoyl-2-oleoylphosphatidylcholine (POPC) lipids as compared with a POPC/2-oleoyl-1-palmitoyl-*sn*-glycero-3-phosphocholine (POPG) (9:1) lipid mixture used in the experimental study by Aimon et al. (62).

For the unbiased study of spontaneous membrane curvature, we chose to study finite membrane patches, i.e., lipid bicelles. The system size of a lipid bicelle surrounded by water is substantial. Therefore, this study additionally aimed at validating a coarse-grained simulation setup in the prediction of protein-induced membrane curvature.

A plain POPC membrane was chosen for all simulations to avoid effects due to inadequate sampling of the lipid distribution in vicinity of the channel as well as curvature-modifying effects from asymmetrical lipid distributions enabled by free lipid diffusion along the bicelle rims.

#### Coarse-grained system setups and parameters

Starting from the readily available crystal structures of AQP0 (PDB: 2B6P; (64)) and KvChim (PDB: 2R9R; (67)), the functional tetrameric assembly of both proteins was generated by multiplication around the crystal axes using PyMol (68). For KvChim, the co-crystallized, cytosolic β subunit (residues 32–144) was deleted for computational efficiency. Therefore, each monomer consisted of residues 145–417 for KvChim and residues 2–242 for AQP0. Subsequently, the systems were transferred into CG representation within the MARTINI 2.2 force field (69,70). Stabilizing ”RubberBand” restraints were applied onto backbone-representing beads using a force constant of 500 kJ/mol/nm^2^, which decays within a cutoff of 0.9 nm at a decay power of 6 and a decay factor of 3 (compare Gahbauer et al. (71)).

Both proteins were inserted into a POPC bilayer employing the *insane* protocol (72). Lipids with a distance of more than 9.25 nm from the respective protein's center of mass were deleted. The resulting finite, circular POPC bilayer patches were solvated at a concentration of 0.15 M NaCl in standard MARTINI water (73). A control system containing a protein-free finite, circular POPC bilayer was additionally prepared (POPC control; compare Table 1). The rectangular simulation box of 30 × 30 × 22 nm contained 169,330 CG particles for the POPC control system (832 POPC lipids), 167,590 particles (661 POPC lipids) for KvChim, and 169,696 particles (732 POPC lipids) for AQP0. After minimization (steepest descent, 500 steps) all systems were shortly equilibrated at increasing integration time steps with position restraints applied on all backbone beads (10,000 steps for dt = 1 fs, 2 fs, 5 fs, 10 fs; 50,000 steps for dt = 20 fs). Ten independent simulations per system (AQP0, KvChim, and POPC) were started for 2 μs each at 310 K and 1 bar using the following parameters: the electrostatics was computed using shifted potentials between 0.0 and 1.2 nm, with a relative permittivity constant $\varepsilon_{r}=15$. van der Waals interactions were shifted to zero between 0.9 nm and 1.2 nm. The temperature of 310 K was kept constant by the v-rescale algorithm (74) with a time constant $\tau_{T}=1$ ps. The pressure was kept constant isotropically at 1 bar using the Berendsen barostat (75), with a coupling time constant $\tau_{p}=4$ ps and a compressibility of $4.5\cdot 10^{-5}$ bar^−1^.

**Table 1:** Lipid (Bicelle) simulation systems

System	# (Lipids)	# (Water)	# (Atoms)	Sim. Time
${\text{POPC control}}^{\text{CG}}$	832	154,632	169,330	10 × 2 *μ*s
$\text{AQP}0^{\text{CG}}$	732	154,504	169,696	10 × 2 *μ*s
${\text{KvChim}}^{\text{CG}}$	661	153,109	167,590	10 × 2 *μ*s
${\text{POPC}}_{\text{infinite}}^{\text{CG}}$	3,042	103,272	145,092	2 *μ*s
$\text{AQP}0^{\text{AA}}$	732	272,681	933,873	0.2 *μ*s
${\text{KvChim}}^{\text{AA}}$	661	272,925	926,691	0.2 *μ*s
${\text{KvP}}_{\text{pos}.\text{restr}.}^{\text{AA}}$	661	274,866	932,498	0.2 *μ*s
$\text{KvP}-\text{MD}100_{\text{pos}.\text{restr}.}^{\text{AA}}$	661	196,422	696,734	0.1 *μ*s
${\text{KvU}}^{\text{AA}}$	661	274,822	932,366	0.2 *μ*s
${\text{POPC}}_{\text{infinite}}^{\text{AA}}$	338	8,472	70,708	0.1 *μ*s

Given are the number of lipids, the number of atoms, the number of water molecules, and the simulation time. Systems are labeled as coarse-grained (CG) systems or all-atom (AA) systems. Studied were lipid bicelles at CG resolution with POPC only (POPC control), with the unrestrained voltage-gated potassium channel (KvChim), and with aquaporin protein (AQP0) embedded within the POPC bicelle. CG simulations were complemented by AA simulations of the lipid bicelle systems.

As a reference system, a periodic (infinite) POPC bilayer was prepared with 3,042 POPC molecules (box size 30 × 30 × 18 nm). Parameters were chosen as described above (except for semi-isotropic pressure coupling), and the simulation was run for 2 *μ*s. All simulations were prepared and performed using the GROMACS simulation package (v.4.6.5) (76,77).

#### Atomistic system setups and parameters

The coarse-grained simulations were corroborated by AA simulations. The atomistic KvChim (AQP0) system is based on a 50 ns coarse-grained simulation of KvChim (AQP0) with position restraints (force constant 1,000 kJ/mol/nm^2^) on all beads of the protein. The simulation systems (proteins and lipids) were transferred back to atomistic resolution (employing the AMBER99SB-ILDN force field (78,79) and the SLipids extension for the lipids (80,81)) using the *initram* protocol (82) (see, e.g., (83) for an assessment of different protein-lipid force fields).

For both membrane proteins, the crystal structure was fitted onto the backmapped protein structure (KvChim RMSD=0.097 nm; AQP0 RMSD=0.128 nm). The resulting bicelle structures were transferred to a rectangular simulation box of size 25×25×15 nm, minimized, and solvated with TIP3P (transferable intermolecular potential 3P) water (84,85) at an ion concentration of 0.15 M NaCl. The final AA systems contained more than 900,000 atoms (661 and 732 POPC lipids for KvChim and AQP0, respectively). Position restraints on all heavy atoms of the protein were applied for 200 ps, with protein coordinates able to adapt to changing box vectors. The production run had a length of 200 ns. Long-range electrostatic contributions were calculated using the particle-mesh Ewald method (86) with a real-space cutoff of 1 nm and a Fourier spacing of 0.12 nm (3D, fourth order). van der Waals interactions were cut off at 1 nm. Temperature at 310 K was kept constant by the v-rescale algorithm with a time constant of $\tau_{T}=0.1$ ps (74). Isotropic pressure coupling at 1 bar was achieved by application of the Parrinello-Rahman barostat (87,88) at a $\tau_{p}=4$ ps and a compressibility of $4.5\cdot 10^{-5}$ bar^−1^.

For comparison, a position-restrained, atomistic KvChim system (“${\text{KvP}}_{\text{pos}.\text{restr}.}^{\text{AA}}$”) was studied within an identical box (25 × 25 × 15 nm; 932,498 atoms). Restraining potentials (force constant 1,000 kJ/mol/nm^2^) were applied to all heavy atoms of the protein based on the crystal structure, rendering the protein inflexible. Simulation parameters were chosen identical to the unrestrained KvChim system. The production run was performed for 200 ns. A second restrained system based on a snapshot of the KvChim system after 100 ns unrestrained equilibration (“$\text{KvP}-\text{MD}100_{\text{pos}.\text{restr}.}^{\text{AA}}$”) was prepared (backbone root-mean-square deviation [RMSD] = 0.195 nm of pore region to starting structure). The system was run for 100 ns (25 × 25 × 11 nm; 696,734 atoms). To evaluate for the influence of the positive charge of KvChim on lipid binding, a system with neutralized charges at the cytosolic leaflet was prepared. The amino acids Arg147, Arg163, Lys247, Arg305, Lys308, Lys318, and Arg322 were neutralized. The simulation was run for 200 ns (system “KvU^AA^”; see Table 1).

For additional reference, we used a 100 ns AA simulation of a preequilibrated (periodic infinite) POPC lipid bilayer (338 POPC molecules) solvated in 0.15 M NaCl (70,708 atoms; compare Table 1). The system was set up using the AMBER99SB-ILDN force field (78,79) with the SLipids extension (80,81). Parameters were selected identical to the above-described bicelle simulations.

All atomistic simulations were prepared and performed using the GROMACS simulation package (v.4.6.5) (76,77).

### Calculation of curvature

The local curvature was analyzed based on the mapping of the bicelle structure on a grid and the calculation of the first and second fundamental form (tool *g_lomepro* (89)). In addition to the grids of the extracellular and of the cytosolic lipid leaflets, the here-adapted version adds a third layer of grid points, defined as the mean positions between the grid points of the extracellular and cytosolic leaflets. Thereby, rather a mean curvature of the lipid bicelle is obtained than a monolayer curvature. Grid points in regions occupied by protein are by default defined as the mean between the highest and lowest membrane normal positions of the selected analysis group (e.g., lipid headgroups), potentially yielding artifacts in curved bicelles. To compensate, grid points were adapted here to be (dynamically) defined by the center of mass of a selectable group of atoms, e.g., of the protein backbone.

Only grid points (= local curvature values) close to the protein (or bicelle center) were considered for further analysis, i.e., only grid points at a distance of 2 nm–5.5 nm from the KvChim center of mass (AQP0: 3 nm–6 nm). Data points outside this region were omitted from further analysis. For pure POPC bicelles, only grid points within 5.5 nm of the bicelle center of mass were considered. The local curvature values were averaged over time windows. Distance-dependent local spontaneous curvature values were retrieved for circular rings around the studied membrane proteins (2 nm–3 nm, 3 nm–4 nm, 4 nm–5 nm, and 5 nm–6 nm relative to the respective protein's center of mass).

The curvature was calculated for 10 ns (20 frames) windows, resulting in 200 consecutive curvature values per 2 μs CG simulation. A resolution of 38 × 38 grid points was chosen; PO4 beads of POPC were used to define the grid. Inclusion of protein atoms was enabled at a precision of 3 nm (compare Fig. 2 in (90)). The central pore domains of both proteins (AQP0: residues 2–263; KvChim: residues 310–417) were used to determine the protein positions. The protein center of mass for the central grid was analyzed using residues 2–241 and residues 310–417 for AQP0 and KvChim, respectively. Omitting the first 100 ns for equilibration effects, curvature histograms were computed over ten simulations (1,900 values) per CG setup with a binning size of 0.005 nm^−1^. A non-linear least squares fit assuming a Gaussian distribution was taken to estimate the expectation value for the (protein-induced) membrane curvature.

For the atomistic simulations, the curvature was as well calculated on a 38 × 38 grid, however for time windows of 1 ns length (including 100 frames). POPC phosphorus atoms were used to define the grid. For KvChim (AQP0), a precision cutoff of 1.5 nm was used. The initial 50 ns of simulation time were omitted from the analysis to exclude equilibration effects, i.e., the curvature analysis was performed on 150 windows, using a binning size of 0.005 nm^−1^.

## Results

### Structural stability of bicelle-embedded proteins

Both investigated membrane proteins, i.e., the water channel AQP0 and the voltage-gated potassium channel KvChim, kept conformational stability in the AA simulations without larger intra-domain rearrangements either in their central tetrameric pore domains or in the voltage-sensing (VS) domains of KvChim as seen in the RMSD: the tetrameric region of AQP0 (residues 2–241) maintained a high stability with an RMSD value of ≈0.17 nm in the 200 ns AA molecular dynamics (MD) simulation as compared with ≈0.3 nm for the TM region of KvChim (residues 145–417; Fig. S1 *A*). This notably larger structural change for KvChim as compared with AQP0 is, however, largely due to the orientational flexibility of the four VS domains as reflected by the lower RMSD values for the central pore domain (residues 310–417) and the individual VS domains (residues 145–309; data not shown) with ≈0.20 nm and ≈0.23 nm, respectively.

For the CG simulations conducted here (MARTINI representation (69,70,73)), the proteins were subjected to *RubberBand* potentials (91) (see Materials and methods section). For AQP0, this additional restraining potential kept the RMSD at ≈0.25 nm within 2 *μ*s of simulation time (Fig. S1 *B*). For comparison, the RMSD increases up to ≈0.6 nm without *RubberBand* potentials. Thus, the parameters chosen in this work (see Materials and methods) allow for structural fluctuations comparable to values obtained in AA simulations. Accordingly, all CG simulations of both AQP0 and KvChim reported in the following were subject to the stabilizing *RubberBand* potentials. Structural deviations of the full coarse-grained KvChim tetramer structures (residues 145–417; backbone beads) quickly stabilized at RMSD values of 0.4 nm–0.5 nm, with smaller deviations of 0.2 nm–0.3 nm and 0.15 nm–0.25 nm for the pore domain (residues 310–417) and VS domains (residues 145–309), respectively, similar to the atomistic simulations. However, the positional flexibility of the VS domains was enhanced in CG simulations as compared with the atomistic simulations.

In summary, the tertiary structures of the individual α-helical domains show high structural stability, both at atomistic and coarse-grained resolution. Comparable levels of CG RMSD values to atomistic results suggest a matching degree of *allowed* structural flexibility. The VS domains show notable whole-body movements with respect to the central pore domain as reflected by the small intra-domain RMSD values as compared with the full protein deviations.

### Protein cross-sectional area across the membrane

**Figure 1:**
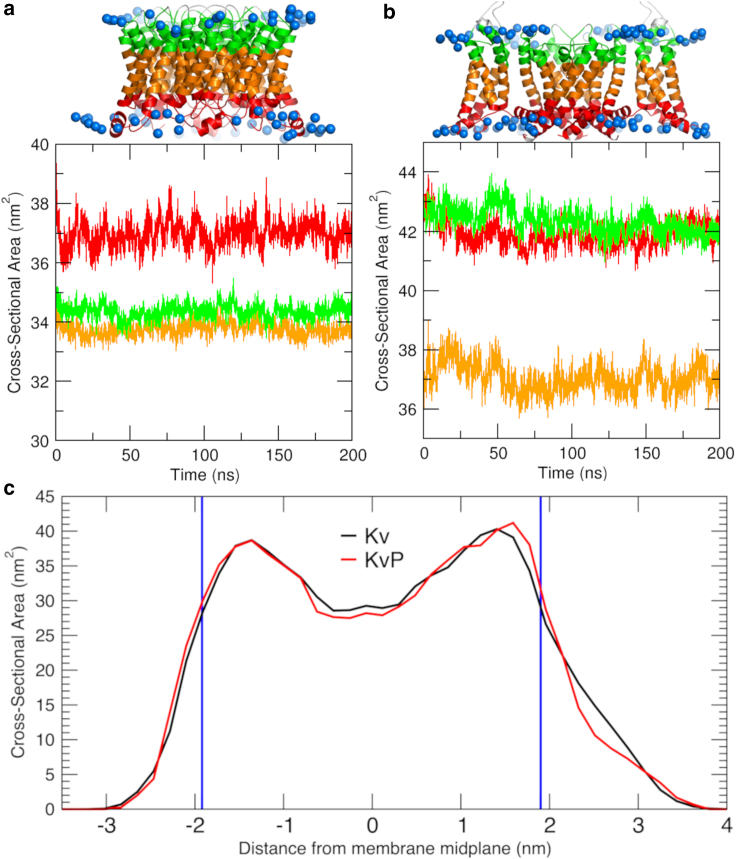
Protein cross-sectional area of AQP0 and KvChim (bicelle systems). Protein cross-sectional area as a function of simulation time and respective mean values. (*A*) Progression of cross-sectional area for different AQP0 segments along the membrane normal is shown (see inset with protein structure and selected segments along the membrane normal; intracellular part in *red*, membrane core region in *orange*, and extracellular protein side in *green*). (*B*) Progression of cross-sectional area for corresponding KvChim segments is shown. Coloring is as in (*A*). (*C*) Fine analysis of cross-sectional area along the membrane normal for KvChim (*black*) and KvP (*red*) is shown, and the locations of interacting POPC headgroups are marked by *blue* lines. To see this figure in color, go online.

The protein cross-sectional area along the membrane normal may affect the local bending of the surrounding lipid membrane, i.e., it may induce a (local) spontaneous membrane curvature. The membrane-embedded parts of the studied proteins were divided into an extracellular interfacial part (marked green in Fig. 1, *A* and *B*), the membrane hydrophobic core region (orange), and the intracellular membrane interfacial protein section (red). The occupied cross-sectional area of the protein for these three regions was analyzed after projection of the respective protein atoms of each region onto the membrane lateral plane (see Supporting material for details). Coarse-grained systems were not considered, since the restraining potentials impede larger conformational rearrangements.

The cross-sectional areas at different membrane positions yield a simplified view on the protein architecture: apart from equilibration effects during the initial 10 ns, the cross-sectional area of AQP0 at the intracellular side is ≈3 nm^2^ larger as compared with the membrane core and the extracellular side (Fig. 1 *A*). These segments were chosen such that lower and upper segments represent the lipid-headgroup-interacting regions of the protein (see Fig. 1, *A* and *B*, insets). AQP0 forms a slightly wedge-like-shaped protein, with the broader end positioned at the intracellular side, i.e., in the lower monolayer of the bicelle. In turn, KvChim displays an hourglass-shaped architecture, with a central cross-sectional area smaller by ≈5 nm^2^ as compared with the membrane interface regions of the channel.

An additional, finer analysis of the cross-sectional area of KvChim along the membrane normal is depicted in Fig. 1 *C*: in general, the hourglass shape could be confirmed; however, new structural details can be observed. The gap between the lipid-exposed regions of the protein and the membrane hydrophobic core was found to be further increased, with comparably small protein cross-sectional areas near the membrane center of ≈29 nm^2^ (KvChim; ≈28 nm^2^ for restrained KvP system; see Materials and methods) within the analyzed 50 ns–200 ns timeframe. Furthermore, a slightly asymmetric form of KvChim is seen with an increased maximum of the cross-sectional area in its extracellular half of ≈40 nm^2^ as compared with ≈38.5 nm^2^ for the intracellular half.

### Protein-induced membrane curvature in finite lipid bicelles

The local spontaneous curvature induced by AQP0 and KvChim was analyzed for the channels embedded in phosphatidylcholine bicelles, for comparison both at AA resolution and at coarse-grained resolution. The AA simulations of the wild-type proteins were complemented by studies of KvChim with restrained structures (KvP and KvP-MD100; see also below), to evaluate the molecular driving forces for the induction of curvature.

**Figure 2:**
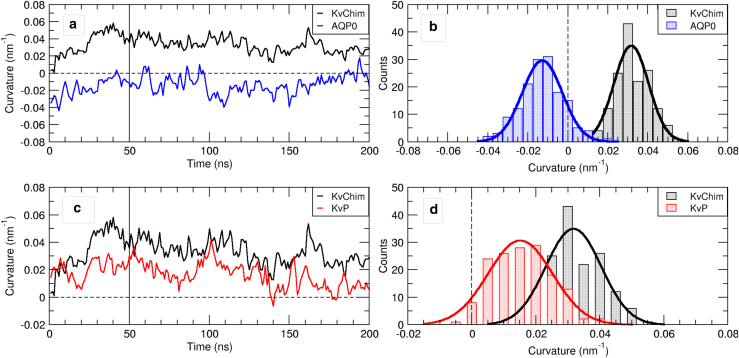
Protein-induced membrane curvature in atomistic simulations (bicelle systems). (*A* and *C*) Time evolution of curvatures in atomistic systems. KvChim is shown in both panels for comparison (*black*). Dotted line is the zero axis, and straight vertical line marks the 50 ns equilibration period. (*B*) Distribution of curvature values for AQP0 and KvChim is shown, excluding the equilibration time. The maxima of the fitted Gaussian distributions (non-linear least-squares fit) was taken as the induced curvature: *H*_AQP0_ = – 0.013 nm^−1^; *H*_KvChim_ = 0.032 nm^−1^. (*D*) Comparison of KvChim to KvP. KvChim is shown for comparison (*black*). *H*_KvP_ = 0.015 nm^−1^. To see this figure in color, go online.

Results of the atomistic simulations displayed in Fig. 2 reveal a pronounced positive curvature induced by the potassium channel (0.032±0.08 nm^−1^) and a negative membrane curvature for bicelles with embedded AQP0 (−0.013±0.10 nm^−1^). These curvature values were averaged over membrane regions at a distance of 2 nm–5.5 nm from the center of mass (COM) of KvChim and 3 nm–6 nm for AQP0. Remarkably, these two curvature estimates retrieved from relatively simple, unbiased MD simulations are in excellent agreement with the experimentally determined values of Aimon et al. (62), both for KvChim (Aimon: KvAP, ≈0.04 nm^−1^) and Aquaporin 0 (Aimon: <0.02 nm^−1^). It has to be noted, however, that the MD simulations in addition yield the direction of induced membrane curvature that was not accessible in experiment.

The curvature analysis of two restrained KvChim bicelle simulation systems—one with KvChim restrained in the initial, crystal-like conformation (KvP; Fig. 2, *C* and *D*) and one with KvChim restrained in a conformation after 100 ns of unrestrained AA MD simulation (KvP-MD100; data not shown)—reveals that even small structural changes may have a strong influence on the induced spontaneous curvature. The average induced curvature for KvP was reduced to 0.015 nm^−1^, although it increased to 0.043 nm^−1^ for KvP-MD100. The curvature of these restrained systems shows fluctuations comparable in magnitude to the unrestrained channel (Fig. 2, *B* and *D*). This points toward an underlying induced spontaneous curvature, established passively around the restrained channel conformation, that is then further enhanced by the protein dynamics.

**Figure 3:**
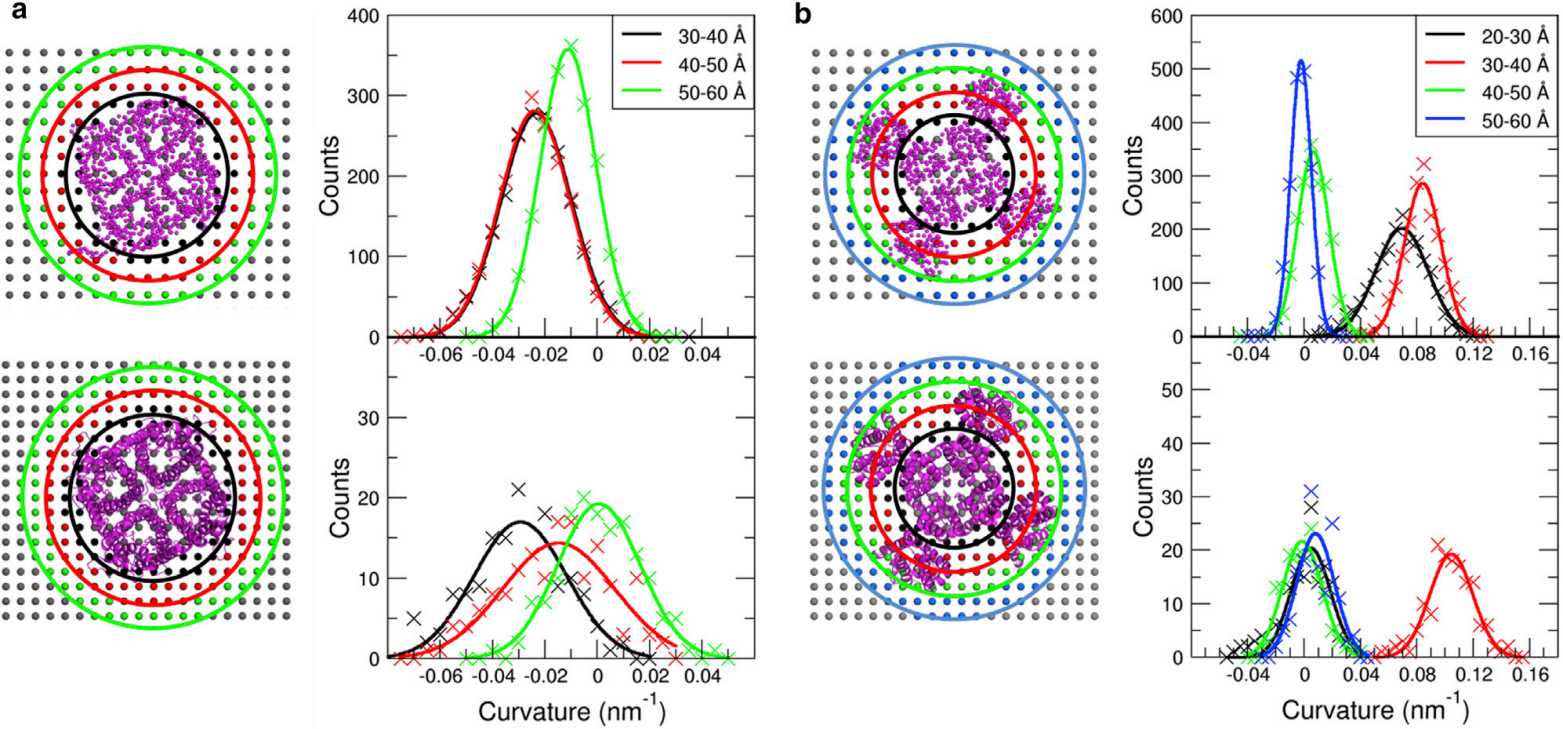
Distance dependency of protein-induced spontaneous curvature (bicelle systems). The protein-induced membrane curvature was analyzed for concentric rings around the protein center of mass. The distributions are based on data between 0.1 *μ*s and 2 *μ*s for coarse-grained simulations (*upper row*) and between 50 ns and 200 ns for the atomistic simulations (*lower row*). (*A*) System with embedded AQP0 is shown; the protein structure with rings indicating the distance selections is shown in the left panel. The curvature distribution maxima were found at 3 to 4 nm: H=−0.029 nm^−1^ (CG: H=−0.024 nm^−1^), 4 to 5 nm: H=−0.014 nm^−1^ (CG: H=−0.024 nm^−1^), and 5 to 6 nm: H=0.00 nm^−1^ (CG: H=−0.011 nm^−1^). (*B*) System with embedded KvChim is shown; the protein structure with rings indicating the distance selections is shown in the left panel. Curvature distribution maxima were found at 2 to 3 nm: H=0.004 nm^−1^ (CG: H=0.07 nm^−1^), 3 to 4 nm: H=0.104 nm^−1^ (CG: H=0.083 nm^−1^), 4 to 5 nm: H=−0.001 nm^−1^ (CG: H=0.006 nm^−1^), and 5 to 6 nm: H=0.009 nm^−1^ (CG: H=−0.002 nm^−1^). To see this figure in color, go online.

To identify possible curvature hotspots in the vicinity of the proteins and quantify the distance dependency of the local induced spontaneous membrane curvature, the curvature was measured as a function of distance from the COM of the respective protein (Fig. 3). The AQP0 bicelle displays a negative curvature within the region 3 nm–5 nm from the protein COM (Fig. 3 *A*, *lower row*), with a vanishing mean curvature at a distance of 5 nm to 6 nm from the protein COM. The largest contribution to the total observed curvature is obtained close to the protein-lipid interface, decreasing with increasing distance from the protein. A different picture can be drawn for KvChim: in the innermost 20–30 Å regime, the curvature distribution displays a maximum close to zero, whereas the curvature distribution in the subsequent 3 nm to 4 nm annulus is drastically shifted in positive direction with a mean curvature of 0.1 nm^−1^ (Fig. 3 *B*, *lower row*). The curvatures obtained for the 4 nm to 5 nm and 5 nm to 6 nm regimes, in contrast, are rather small with mean values close to zero. The 3 nm to 4 nm ring (Fig. 3 *B*, *red*) is located just between the VS domains of KvChim. Interestingly, analysis of the variant KvP reveals comparably large levels of 0.05 nm^−1^ within the first two regimes 2 nm to 3 nm and 3 nm to 4 nm (Fig. S2). In summary, although the local induced curvature in case of AQP0 is most prominent in direct vicinity of the protein structure, in accordance with the wedge-like shape of the tetramer, the membrane curvature induced by KvChim is focused in the VS domain regions.

The results on protein-induced curvature retrieved from atomistic simulations show encouraging agreement to experiment. However, the statistics are limited by the required substantial computational resources for the simulation of AA bicelle systems (see Materials and methods section). In exchange for structural detail, excellent statistics on the protein-induced curvature may be collected, replacing atomistic detail by a coarse-grained description of the bicelle system. To this end, we here employed the MARTINI force field (69,70,73).

**Figure 4:**
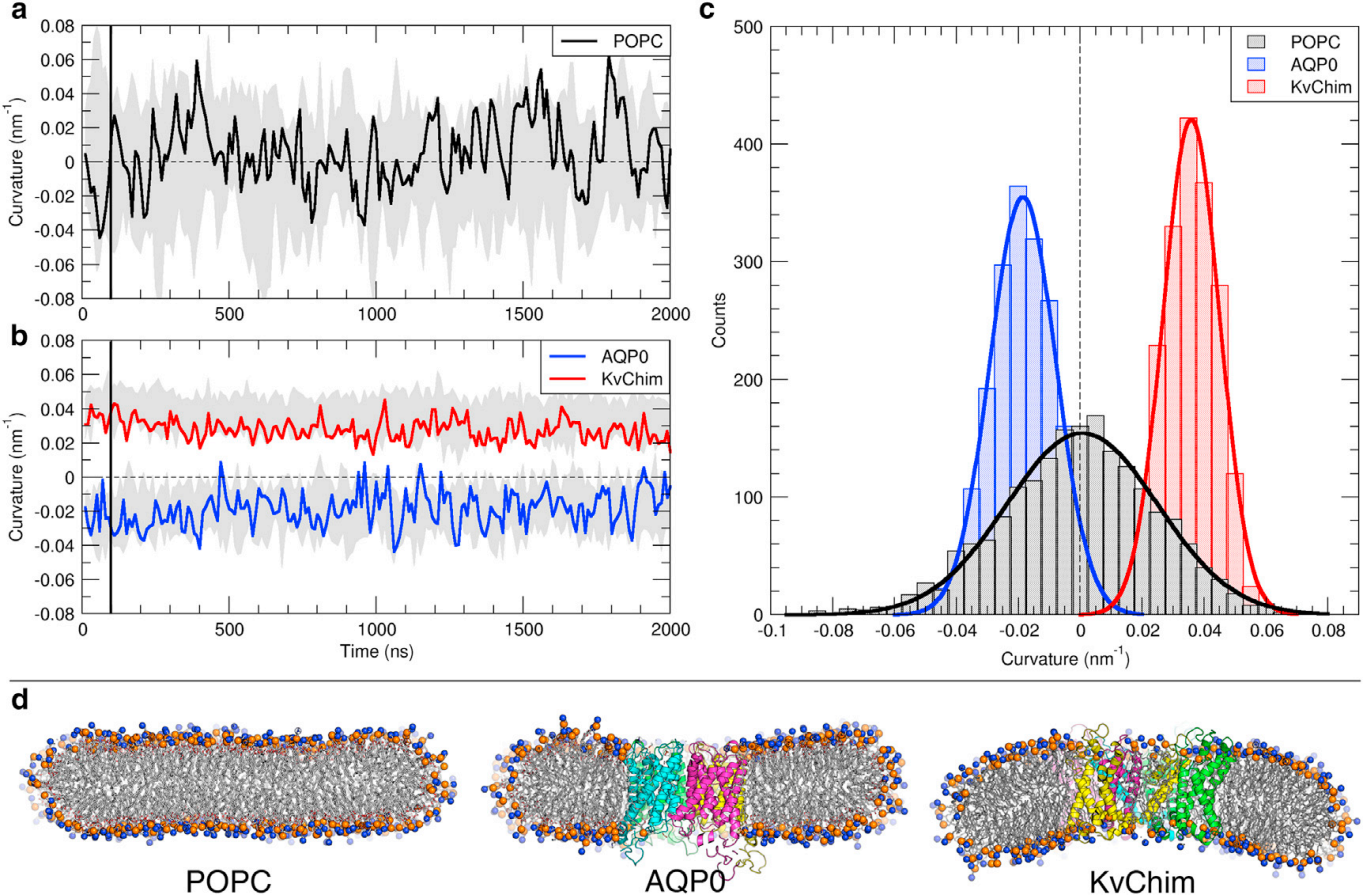
Protein-induced membrane curvature in coarse-grained systems. (*A* and *B*) Fluctuations of the spontaneous curvature of lipid bicelle systems obtained from ten independent coarse-grained simulations (*gray* shaded area). The time evolution of the membrane curvature of replica simulation MD 1 is shown exemplarily (solid line). (*C*) Distribution of bicelle curvature values with and without embedded membrane protein and fitted Gaussian distributions (solid lines) is shown. (*D*) Cut-through bicelles for representative values of the spontaneous curvature are shown. Proteins are shown in cartoon representation and colored by chain. POPC headgroup atoms are shown as spheres; POPC control is at a curvature of 0 nm^−1^, AQP0 at −0.019 nm^−1^, and KvChim at 0.036 nm^−1^. To see this figure in color, go online.

Control simulations of protein-free POPC bicelles in the MARTINI framework show fluctuations between −0.05 nm^−1^ and 0.05 nm^−1^ (Fig. 4, *A*, *gray shaded area*) but no preference for either direction of bending, i.e., a vanishing spontaneous membrane curvature well fitted by a Gaussian distribution centered at 0 nm^−1^ (Fig. 4, *C*, *black*).

In contrast, lipid bicelles with embedded AQP0 show negatively shifted curvature values (Fig. 4 *B*) with a distribution maximum at −0.019 nm^−1^ (Fig. 4 *C*, *blue*). Thus, the curvature deduced from coarse-grained simulations is in very good agreement with the corresponding result from atomistic simulation (−0.013 nm^−1^). Similarly, curvature values retrieved from a bicelle-embedded KvChim generally displayed a positive shift (Fig. 4 *B*, *red*), clearly distinct from the AQP0-induced curvature. The curvature values averaged over ten independent 2 μs-long CG simulations yield an average curvature of 0.036 nm^−1^ (Fig. 4 *C*, *red*), again in very good agreement to results from atomistic simulation (0.032 nm^−1^) and experiments (62). The slightly increased values for the spontaneous curvature within the coarse-grained description are connected to a lowered bending modulus: the bending modulus for an atomistic POPC bilayer was reported to 24.3 $k_{B}T$ (CHARMM36 force field) as compared with 14.1 $k_{B}T$ determined here for a POPC bilayer within the MARTINI force field employing the real-space fluctuation method (92) or, alternatively, to 22.7 $k_{B}T$ by analysis of the fluctuations of lipid orientations (93).

Both embedded channels reduced the fluctuation width of the bicelle curvature to ±0.015 nm^−1^ for AQP0 and ±0.01 nm^−1^ for KvChim, based on the curvature fluctuations (gray shaded areas) with respect to the calculated means. Cuts through the bicelles for representative values of the curvature are displayed in Fig. 4 *D*. In summary, at coarse-grained resolution, we find methodological support as represented by the protein-free reference simulations, with no detectable preference for curvature, as well as protein-induced curvature in levels comparable to atomistic simulations, while also being consistent with the experimental data retrieved from sorting experiments (62). In addition, statistical confidence for the described curvature could be established based on multiple, independent simulation replicas.

The role of protein dynamics for the induced membrane curvature was assessed by additional protein-restrained CG MD simulations carried out both for AQP0 and KvChim: AQP bicelles did not display any change in curvature with respect to the dynamic CG systems, with an observed average negative curvature of −0.02 nm^−1^ and fluctuations of ±0.015 nm^−1^ (data not shown). Thus, the observed wedge-like shape of AQP0 remains the determining membrane-shaping factor. For KvChim in turn, a reduction of the induced curvature by almost 50% upon restraining the structure is observed, similar to the results obtained for atomistic simulations (KvP). Here, the fitted mean curvature was 0.02 nm^−1^ with fluctuations of ±0.01 nm^−^^1^ (10 replica simulations, each of 1 μs length).

Subsequent splitting of the curvature data retrieved from CG simulations into distance-dependent rings shows that the AQP0-induced spontaneous curvature originates largely from lipids between 3 and 5 nm from the protein COM, i.e., close to the protein interface and similar to the AA simulations (Fig. 3 *A*, *upper panel*). These results further support the finding that protein shape is the primary curvature driving force for AQP0. Interestingly, a comparison of POPC positions close to AQP0 from AA simulations with the position of co-crystallized 1,2-dimyristoyl-sn-glycero-3-phosphocholine (DMPC) molecules (see Fig. S3) shows that POPC headgroup positions are shifted with respect to DMPC crystal positions mainly within the intracellular leaflet (Fig. S3, *C* and *D*) to account for the increased acyl chain length.

For KvChim, the overall induced positive curvature is to a large degree dependent on lipids located between the voltage-sensing domains (3 to 4 nm; Fig. 3 *B*, *upper panel*, *red*), however with an increased contribution of lipids in vicinity of the central pore domain (2 to 3 nm) as compared with atomistic simulations. Nevertheless, the KvChim-induced membrane curvature can largely be attributed to the interstitial membrane regions formed by the VS domains, similar to what is observed in atomistic simulations.

### Membrane characteristics in KvChim lipid bicelles

The voltage-gated potassium channel KvChim shows a pronounced asymmetry in protein-lipid binding between the extracellular and the cytosolic leaflets, despite the similar cross-sectional areas of the membrane-spanning channel within both leaflets (compare Fig. 1 *B* and Fig. 1 *C*): in the atomistic MD simulations, 43±2 lipids were found within 0.7 nm distance of the protein backbone in the cytosolic leaflet as compared with only 32±3 extracellular lipids in contact with the channel. This difference was reproduced in the coarse-grained simulations (cytosolic: 34.6±0.2 lipids; extracellular: 27.9±0.2 lipids within 0.7 nm of the protein backbone). In addition, the contact times between lipids and KvChim were significantly longer within the cytosolic membrane half (≈20 ns versus ≈10 ns within the extracellular leaflet).

**Figure 5:**
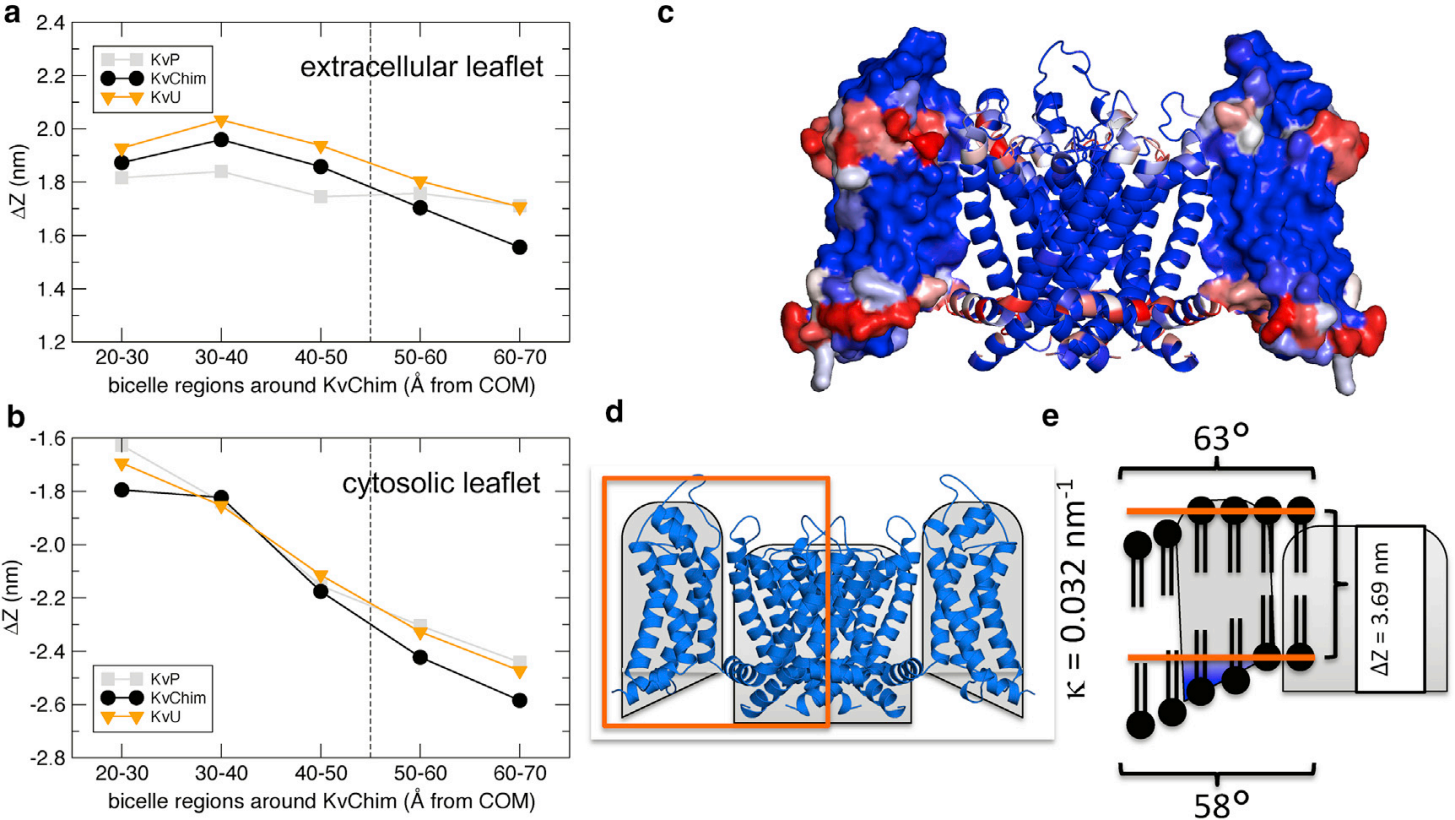
Voltage-sensing domains induce membrane curvature. (*A* and *B*) POPC headgroup membrane normal positions (*z*) with respect to the center of mass (COM) of the pore domain of KvChim (residues 310–417, backbone atoms). Plotted are the differences Δz between the COM of the pore domain and the COM of the phospholipid headgroups of the extracellular (*A*) and of the cytosolic leaflet (*B*), respectively, within defined 1-nm-distance segments from the COM of the pore domain. Results are shown for the atomistic MD simulation of KvChim, for the position-restrained potassium channel (“KvP”), and for a partially uncharged KvChim (“KvU”; compare Materials and methods). (*C*) KvChim structure color coded according to the frequency of binding of lipid phosphate groups to channel amino acids (distance <0.7 nm) determined from coarse-grained simulation is shown. (*D*) Crystal structure of KvChim (*blue*) with simplified, schematic structure shown as *gray* background. (*E*) Schematic summary of how KvChim induces membrane curvature predominantly by the cytosolic part of the voltage-sensing domains is shown. To see this figure in color, go online.

The membrane normal positions of the lipid headgroups within concentric regions around the channel suggest that the overall channel-induced positive membrane curvature can largely be attributed to the tight interaction of lipids with the cytosolic domain of the voltage-sensing domain of KvChim: the cytosolic lipids placed between the voltage-sensing domains are shifted downwards by 0.4 nm within 3–5 nm distance to the KvChim COM as compared with the lipids proximal to the central pore domain (Fig. 5, *A* and *B*). In contrast, the averaged lipid normal position did not change substantially for the extracellular lipids close to the channel. These lipid shifts did not critically depend on the protein charge or conformational changes, atomistic simulations with neutralized cationic amino acids within the cytosolic part of the channel (system “KvU”) or with restrained backbone dynamics (system “KvP”) showed similar trends (Fig. 5, *A* and *B*). The membrane curvature is instead imposed by the particular shape of the voltage-sensing domains that bear an inclined interface at the cytosolic boundary of the potassium channel (Fig. 5, *C*–*E*). These domains provide a sticky environment for phospholipids, thereby imposing a membrane tilt, i.e., curvature within the VS interstitial region. Noteworthy, the POPC lipids between the voltage-sensing domains sample similar positions as were observed for co-crystallized POPG molecules (67) (see Fig. S4). Together with the experimental finding of a similar curvature-dependent enrichment of KvAP for lipid mixtures containing either phosphatidic acid or phosphatidylglycerol (62), this suggests that the curvature effect of voltage-sensitive potassium channels is likely not due to binding of specific lipids.

## Discussion

Here, using lipid bicelle morphology setups in both atomistic and coarse-grained molecular dynamics simulations, we report local induced membrane curvature values retrieved from simulations of two TM model proteins (AQP0 and the voltage-gated potassium channel KvChim) embedded in a POPC bicelle in excellent agreement with experimental data (62). It has to be noted, however, that the giant unilamellar vesicles used in the experiments additionally contained 10% anionic lipids (POPG).

Importantly, the in silico approach additionally predicts the *direction* of membrane deformation at full atomistic resolution. In other words, the lipid bicelle simulation system is established as a fruitful approach for the in-depth analysis of protein-induced spontaneous membrane curvature. The recently published possibilities of resolution transfer between atomistic and coarse-grained representations also employed in this work further expands the possibilities of lipid bicelle systems (82).

The shape of a TM protein, especially the lipid-facing surface, provides the most direct link between protein structure and spontaneous membrane curvature (94). The structures of AQP0 and KvChim used in this study, although showing distinct structural features, do not display easy to identify inclined lipid interfaces as opposed to, for example, the potassium channel KcsA (95,96). KvChim adopts a relatively complex shape, with four symmetrically attached VS domains flanking the *α*-helical central pore, but without showing tilted interfaces along the membrane normal. On the contrary, KvChim was shown to be symmetrical along the membrane normal by cross-sectional area analyses. The AQP0 tetramer, without additional attached domains, exhibits a simple *α*-helical barrel-like structure but in fact was found to display a wedge-like increase of cross-sectional area in the cytosolic leaflet.

Evidenced by this increased cross-sectional area of ≈3 nm^2^ at the cytosolic membrane interface, cytosolic leaflet lipids close to AQP0 (3 to 4 nm) are displaced, giving rise to negative curvature stress (50). This idea is further supported by an increased, local *negative* curvature in the same 3- to 4-nm region of the AQP0 bicelle, at both coarse-grained and atomistic resolution. Thus, AQP0-induced curvature was found to be caused by specific lipid interactions and displacement at the tetrameric protein surface. The possibility of compensation of the asymmetric cross-sectional area by suitable bound lipid species (97) in the extra- or intracellular leaflet, with subsequent changes in the curvature preference, has to be noted, as, for example, observed in the original, planar crystals including DMPC lipids (64).

Structural observables, as well as simple headgroup position assessment of lipids in KvChim vicinity, demonstrate a crucial role of the attached VS domains in membrane remodeling by KvChim. Lipid headgroups of the cytosolic leaflet were gradually shifted along the membrane normal with increasing distance from the central pore (≈0.6 nm shift between 2 nm and 6 nm radius from the central pore). In contrast, lipid headgroup positions of the extracellular leaflet were hardly shifted along the membrane normal within this region around the channel. Accordingly, the bilayer thickness close to the central pore domain (2 to 3 nm) was reduced as compared with protein-distal bicelle regions (data not shown), i.e., the membrane compressed, an observation which is known in literature and discussed to focus the electrical field around the ion channel (98). In consequence, the curvature stress induced by the asymmetric lipid positions between both leaflet interfaces results in a positive membrane curvature, even in simulations with a restrained protein conformation at both coarse-grained and atomistic resolution. Our simulations rule out that KvChim induces a nearly as strong curvature as reported from the analysis of diffusion experiments for KvAP that suggested an as large curvature as 0.16 nm^−1^ (65).

It has to be noted that the specific curvature-inducing interface of KvChim is not reflected in the cross-sectional protein area, where KvChim displays a largely symmetrical, hourglass-like shape. Noteworthy, the POPC membrane thickness within the bicelle system is overall increased as compared with infinite membrane systems for the atomistic system from 3.7 nm to 3.8–3.9 nm in vicinity of KvChim and 4.0 nm for the region 5 to 6 nm from the channel COM. The overall increased membrane thickness in the bicelle systems is related to the increased line tension at the bicelle rims.

In literature, a tendency for increased VS domain motility has been reported for the related potassium channel Kv1.2 (98, 99, 100) in atomistic simulations and was discussed to be connected to reequilibration from non-native crystal packing. However, possible effects of the VS domain on the surrounding membrane were not discussed. Here, we find that the spontaneous curvature contribution by asymmetrical membrane compression at the protein-lipid interface was further enhanced in unrestrained MD simulations of KvChim by repositioning and reorientation of the VS domains. Already slight relative rearrangements influenced the surrounding lipid bicelle, in particular since ≈80% of all lipids interacting with KvChim are in the vicinity of the mobile VS domains. This notion was rationalized by simulations of specifically restrained KvChim structures: although the first system (KvP, restrained crystal structure) displayed reduced but still recognizable curvature in positive direction, highlighting the effect of lipid organization along the interface only, the second system (KvP-MD100) displayed continuously high levels of curvature, with slightly reoriented VS domains frozen at positions after 100 ns of unrestrained MD, therefore depicting the impact of structural displacement on the surrounding bicelle. Such a dependency of the induced positive spontaneous curvature on the specific positioning of the VS domains hints to a mechanical coupling of voltage-dependent K^+^ channels to the lipid environment. Most interestingly, a high tension sensitivity was reported for different voltage-dependent potassium channels (101).

At this point, it has to be noted that the protein crystal structure of KvChim includes an additional, large tetrameric intracellular part in the crystal, named *β* subunit, that is connected to the protein at residue 145 of each VS domain. However, previous simulation studies of KvChim with omitted *β* subunit and of the homologous potassium channel Kv1.2, similarly omitting the cytosolic T1 domain, validated that the structural stability of the remaining protein is not impaired (100,102). Still, due to the importance of VS domain dynamics for observed curvature in the bicelle system, extended simulations including the *β* subunit might be of interest for future investigations.

## Conclusions

In conclusion, the work presented in this study highlights the importance of structural dynamics in hindsight of protein-induced spontaneous membrane curvature. Although different proteins have been reported to be engaged in curvature generation or curvature-sensing processes, such as BAR-domain molecules, displaying shapes matching their specific function (and target curvature), this picture remains mostly unclear in the case of TM proteins partaking in curvature-relevant processes. Although recent studies reported individual TM proteins to be affected or sorted by the presence of specific cellular curvature (61,65), most resolved TM protein structures are not discussed with regard to the preferred environmental, or spontaneously self-induced, curvature. However, it seems unlikely that only a minuscule fraction of the proteins on crowded cellular membranes are receptive to the plethora of possible curvatures observed in biological systems on the micro- and macroscopic scale. Based on the observed curvature difference of the model proteins AQP0 and KvChim, caused by their difference in structure, organization, and dynamics, we suggest that TM proteins, first, can display notable spontaneously induced local curvature, even if structural analyses show no strikingly tilted protein shape, and, second, that TM proteins might be prone to ongoing dynamic regulation of their preferred inherent curvature, due to structural dynamics induced or modified by smaller molecules or specific lipid species. The induced spontaneous curvature is confined to a region extending by up to ≈2 nm from the protein surface.

Methodologically, lipid bicelle systems appear to be ideally suited to determine membrane protein-induced spontaneous membrane curvature in simulations. Coarse-graining further pushes large-scale studies on the membrane-shaping effect of TM proteins within reach. Appropriate measures to reduce the line tension at the bicelle rims and to avoid lipid diffusion between the leaflets will additionally advance studies on membrane (re)shaping in complex biomembrane mimics.

## Author contributions

C.K. and R.B. designed research, C.K. performed the simulations, and C.K. and M.P. analyzed the data. All authors wrote the manuscript.

## Supporting material

Document S1. Figures S1–S4

Document S2. Article plus supporting material

## Footnotes

Supporting material can be found online at https://doi.org/10.1016/j.bpj.2022.01.029.
