# Supplementary material for: Spontaneous local membrane curvature induced by transmembrane proteins
Source: Biophys J. 2022 Feb 3;121(5):671–83. doi: 10.1016/j.bpj.2022.01.029 (PMC8943716; doi:10.1016/j.bpj.2022.01.029)
Supplement: Document S1. Figures S1–S4 [file mmc1.pdf]

Biophysical Journal, Volume *121*

Supplemental information

Spontaneous local membrane curvature induced by transmembrane proteins

Christoph Kluge, Matthias Pöhnl, and Rainer A. Böckmann

Supplementary Information: Spontaneous Local Membrane Curvature Induced by Transmembrane Proteins

Christoph Kluge[1], Matthias Pöhnl[1], and Rainer A. Böckmann[1,2]

[1]Computational Biology, Department of Biology, Friedrich-Alexander University Erlangen-Nürnberg, Erlangen, Germany

[2]National Center for High-Perfomance Computing Erlangen (NHR@FAU)

[*]rainer.boeckmann@fau.de

January 25, 2022

Calculation of lipid headgroup positions

The tool FATSLiM [1, 2] significantly simplifies the analysis of non-standard membrane morphologies in MD simulations. Lipid bilayer leaflet identification is achieved by geometric reduction of all available lipids and comparison of relative lipid orientations. This approach is successful as long as the compared orientations of the lipids are sufficiently different from another (see online documentation for details [2]). However, the micellar-like boundary of lipid bicelles impedes the automatic leaflet detection based on lipid orientation. Therefore, we here employed a distance-threshold from the embedded membrane protein or the center of mass of the bicelle to dynamically select bicelle lipids excluding the rim lipids. Due to its per-lipid algorithm for thickness calculation based on lipid orientation and inter-lipid distances, the calculation of membrane thickness in FATSLiM becomes independent of the membrane shape.

In preparation of the FATSLiM analyses, the input trajectories of the investigated simulation systems were filtered to reduce high-frequency fluctuations which have been noticed to

impair the correct identification of the bicelle membrane leaflets. This was performed using the lowpass-filter function of the GROMACS tool *g_filter* using filter lengths of 10 ns for the coarse-grained trajectories of AQP0, KvChim variants, and POPC bicelles. For the atomistic simulations a filter length of 1 ns was employed.

The distance thresholds for the exclusion of lipids within the rims of the bicelle were chosen as follows: In coarse-grained POPC bicelle systems, PO4 beads beyond 7.5 nm from the center of mass of all present POPC lipids were excluded. The same threshold of 7.5 nm was applied to KvChim simulations, analyzed as the distance to the center of mass of the central pore (residues 309 - 417). For AQP0, a slightly larger threshold of 8.0 nm could be used due to the comparably large diameter of the protein. Lipid phosphorus atoms were used as analysis group in the all-atom simulations with identical thresholds (AQP0: 8.0 nm, KvChim variants: 7.5 nm).

Artifacts due to lipid diffusion were avoided by re-calculation of all distances, i.e. lipid index groups, for each frame of the (low-pass filtered) trajectory. Important cutoffs of the FATSLiM algorithm were chosen as follows: The cutoff for leaflet identification was kept at its default value of 2 nm, as well as both thickness cutoffs of 2 nm and 6 nm. Throughout the analyses, protein positions were included where possible. Hydrogen atoms were removed for the analysis. Data was further analyzed using BASH scripts and MATLAB [3]. In summary, time-dependent information of the studied observables as a function of distance from the protein was achieved.

Cross-sectional protein area

The protein cross-sectional area along the membrane normal was calculated for the atomistic KvChim and AQP0 trajectories as follows: Protein structures were written from the trajectory every 100 ps in PDB format. For each structure, the periodic box was cut into 30 slices along the membrane normal (z-dimension) with a thickness of 3.688 Å for the fine analysis, and into 7 slices (15.8 Å) for a coarsened description. Protein atoms were filtered according to their Euclidian z-coordinate and assigned to their respective slice. The coordinates of atoms within the respective slice were used as input for the calculation of the lateral cross-sectional area. The area was analyzed using the α-shapes approach [4] at an α-value of 3.6 for the fine analysis, and 2.0 for the three segment analysis. The calculated protein cross-sectional area per slice was collected for all frames as function of time and used for the calculation of mean values.

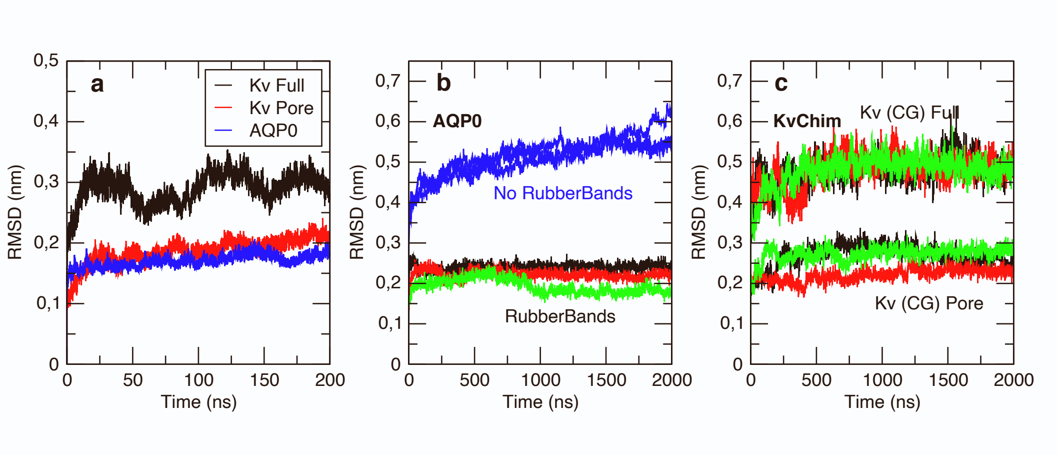

Figure S1: **Root mean square deviation (RMSD) of homo-tetramer membrane proteins AQP0 and KvChim.a**. *RMSD of AQP0 (blue line) and KvChim as a function of simulation time in atomistic simulations (backbone). The RMSD of KvChim is given both for the full structure (black) as well as for the central pore domain only (red, residues 310 - 417).* **b**. *Influence of "Rubberband" potentials on the RMSD of AQP0 in three replica coarse-grained simulations.* **c**. *RMSD of KvChim in three replica coarse-grained simulations separately for the full structure as well as for the central pore domain.*

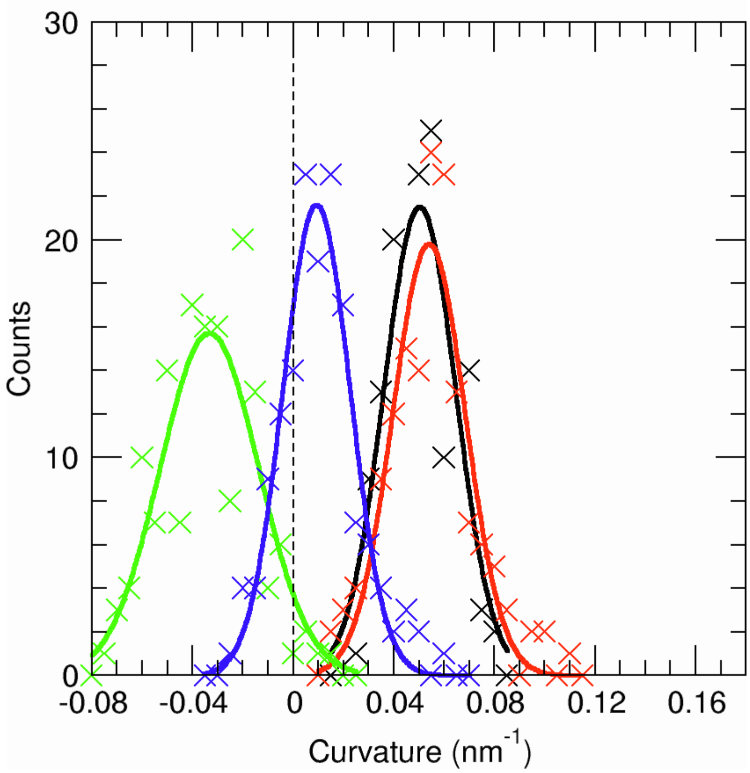

Figure S2: **Distance-dependency of protein-induced spontaneous curvature for position-restrained atomistic simulation of KvChim (KvP).** *The protein-induced membrane curvature was analyzed for for concentric rings around the protein center of mass. The distributions are based on data between 50 ns - 200 ns for different concentric rings around the center of mass of KvChim (black: 20 -30 Å, red: 30 - 40 Å, green: 40 - 50 Å, blue: 50 - 60 Å).*

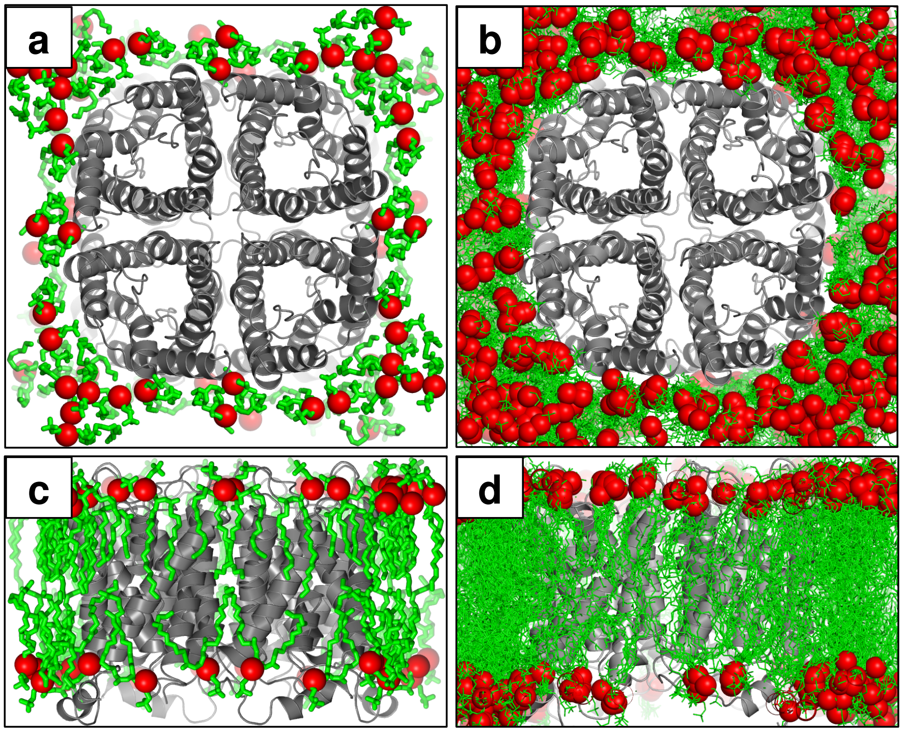

Figure S3: **AQP0 with bound lipids.** *The structure of AQP0 (gray cartoon representation) [5] with co-crystallized DMPC molecules (top view,* **a***; side view,* **c***) is compared with 10 overlayed structures of surface-bound POPC molecules (retrieved from last 10 ns of atomistic MD simulations) for the AQP0^{AA} system (***b***,* **d***).*

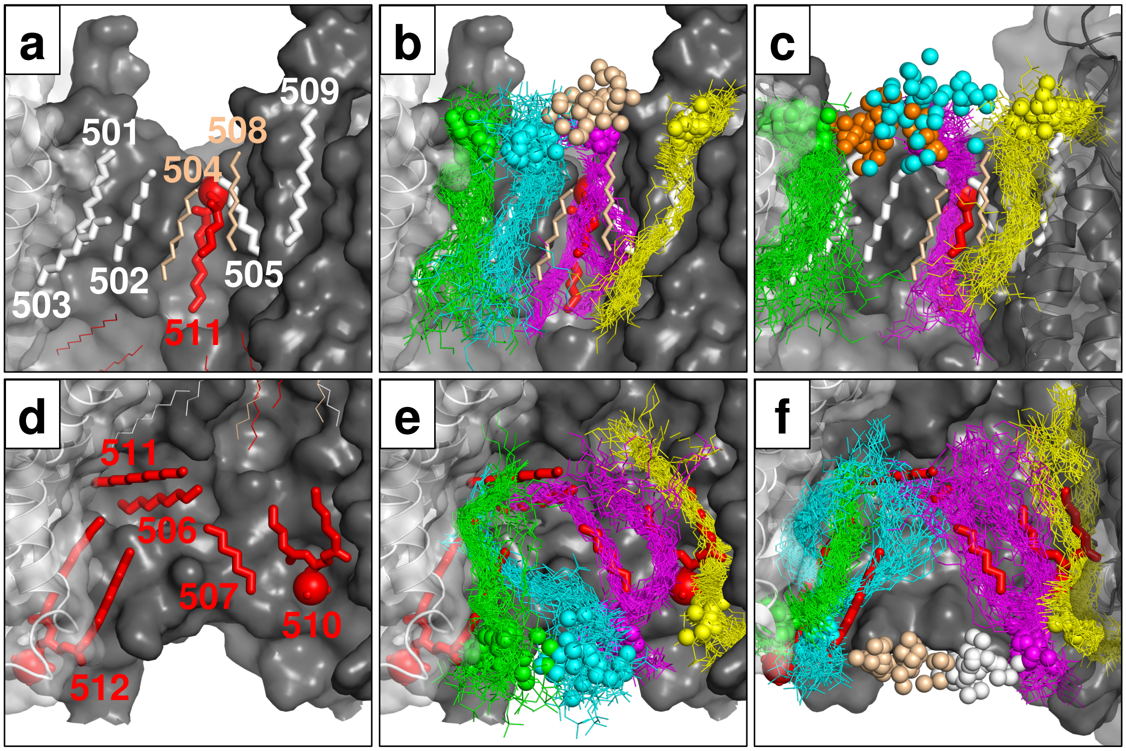

Figure S4: **KvChim with bound lipids.** *The structure of KvChim (gray surface representation) with (selected) co-crystallized POPG fragments [6] within the extracellular (***a***, upper row) and intracellular leaflet (***d***, lower row) is compared with 50 overlayed structures of POPC (retrieved from last 50 ns of atomistic MD simulations) each for the position-restrained* $KvP^{AA}_{pos.restr.}$ *system (***b***,* ***e***) and the free* $KvChim^{AA}$ *simulation (***c***,* ***f***). Shown is the region between two voltage-sensing domains. Most of the POPG fragment positions are well sampled by POPC in the atomistic simulations.*

References

[1] Buchoux, S., 2016. FATSLiM: A fast and robust software to analyze MD simulations of membranes. *Bioinformatics* 33:133–134.

[2] FATSLiM online documentation. `http://pythonhosted.org/fatslim/`.

[3] MATLAB and Statistics Toolbox Release 2014b. The MathWorks, Inc., Natick, Massachusetts, United States.

[4] Edelsbrunner, H., and D. G. Kirkpatrick, 1983. On the shape of a set of points in the plane. *IEEE Trans. Inf. Theory* 29:551–559.

[5] Gonen, t., Y. Cheng, P. Sliz, Y. Hiroaki, Y. Fujiyoshi, S. C. Harrison, and T. Walz, 2005. Lipid–protein interactions in double-layered two-dimensional AQP0 crystals. *Nature* 438:633–638.

[6] Long, S. B., X. Tao, E. B. Campbell, and R. MacKinnon, 2007. Atomic structure of a voltage-dependent K+ channel in a lipid membrane-like environment. *Nature* 450:376–382.
